# Thermodynamics of DNA: heat capacity changes on duplex unfolding

**DOI:** 10.1007/s00249-019-01403-1

**Published:** 2019-11-05

**Authors:** Anatoliy Dragan, Peter Privalov, Colyn Crane-Robinson

**Affiliations:** 1 Institute of High Technologies, Taras Shevchenko National University of Kyiv, Kyiv, 01601 Ukraine; 2 Department of Biology, Johns Hopkins University, Baltimore, MD 21218 USA; 3 Biophysics Laboratories, School of Biology, University of Portsmouth, Portsmouth, PO1 2DT UK

**Keywords:** DNA, Heat capacity, Hydration, Solvent accessible surface area, Surface polarity

## Abstract

The heat capacity change, ΔCp, accompanying the folding/unfolding of macromolecules reflects their changing state of hydration. Thermal denaturation of the DNA duplex is characterized by an increase in ΔCp but of much lower magnitude than observed for proteins. To understand this difference, the changes in solvent accessible surface area (ΔASA) have been determined for unfolding the B-form DNA duplex into disordered single strands. These showed that the polar component represents ~ 55% of the total increase in ASA, in contrast to globular proteins of similar molecular weight for which the polar component is only about 1/3rd of the total. As the exposure of polar surface results in a decrease of ΔCp, this explains the much reduced heat capacity increase observed for DNA and emphasizes the enhanced role of polar interactions in maintaining duplex structure. Appreciation of a non-zero ΔCp for DNA has important consequences for the calculation of duplex melting temperatures (*T*_m_). A modified approach to *T*_m_ prediction is required and comparison is made of current methods with an alternative protocol.

## Introduction

It is well established that the heat denaturation of globular proteins is accompanied by an increase in the heat capacity of the system as a consequence of the hydration of internal hydrophobic resides by weakly bound water molecules having a heat capacity greater than bulk water. In contrast, when polar residues become exposed on protein denaturation, solvating water molecules are more tightly bound than in free solution, so their heat capacity decreases (Makhatadze and Privalov 1990; Privalov and Makhatadze 1992; Spolar et al. 1992; Murphy and Friere 1992; Loladze et al. 2001).

The total heat capacity change, ΔCp(*T*), is frequently formalized in equations of the type:

1 $$ \Delta {\text{Cp}}\left( T \right) \, = \, \varSigma \Delta ({\text{ASA}})^{i} \times \Delta {\text{Cp}}^{i} \left( T \right), $$

where the coefficients ΔCp^*i*^ (*T*) represent the heat capacity change per Å^2^ of surface of defined type *i* and Δ(ASA)^*i*^ is the change (increase) in the accessible surface area of that type that becomes exposed upon unfolding. Coefficients have been derived by several authors for the polar, aliphatic and aromatic surface of proteins although the last two categories are often combined in a single apolar term. According to Makhatadze and Privalov (1995), the heat capacity effect of hydrating the apolar and polar groups of proteins can be expressed by the equation:

2 $$ \Delta {\text{Cp }}\left( {25\;^\circ {\text{C}}} \right) \, = \, 2.14 \times \Delta {\text{ASA}}_{\text{apolar}} - 1.27 \times \Delta {\text{ASA}}_{\text{polar}} , $$

where.ΔASAs represent the increase in accessible surface area of apolar and polar surface.

The heat capacity change, ΔCp, is an important parameter because it represents the temperature dependence of the enthalpy of the process: $\Delta {\text{Cp }} = \delta \left( {\Delta H} \right)/\delta T$. Knowledge of ΔCp, therefore, allows comparison of denaturation enthalpies at a standard temperature for proteins having very variable melting points (Privalov 2012). Equation (2)—but with opposite signs—represents the heat capacity change resulting from the dehydration of internal residues on folding and has also been of value in characterizing protein/DNA association interactions in terms of the interfacial surface area occluded. The observed heat capacity change on forming a protein/DNA complex was separated into the contribution from dehydration of protein surface, calculated for example using Eq. (2), and that from dehydration of the DNA surface to which the protein binds (e.g., Dragan et al. 2003). Application of this protocol to a substantial set of major and minor groove binding proteins led to the derivation of ΔCp^*i*^ coefficients for the dehydration of unit surface area in both grooves of the duplex (Dragan et al. 2019).

## The heat capacity of the DNA duplex

**Fig. 1:**
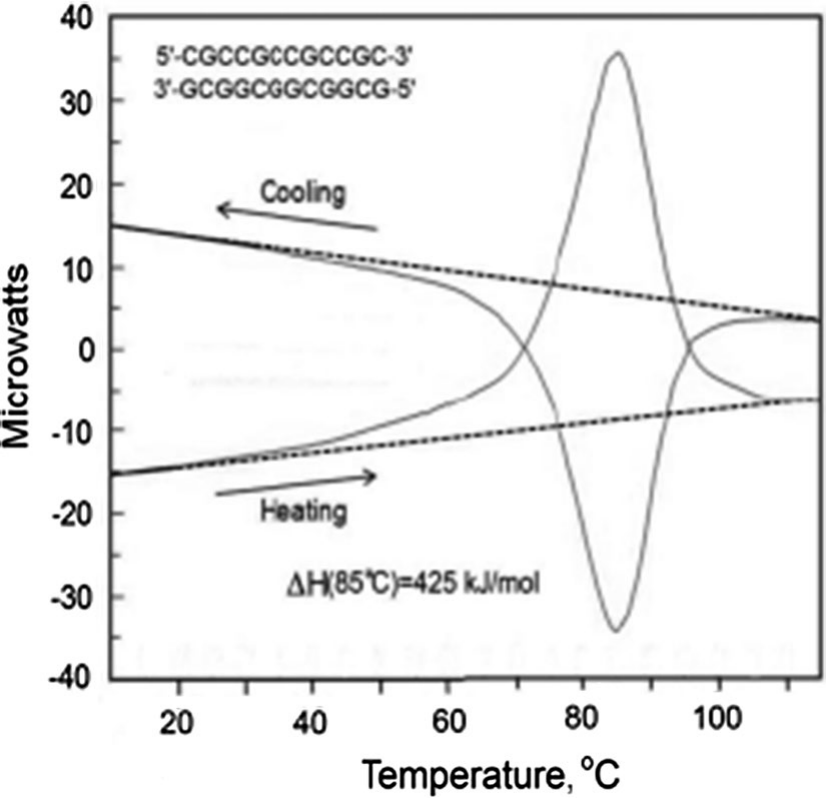
Original DSC recordings of the heat effect on heating and subsequent cooling at a constant rate of 1 K min^−1^ of a 12 bp all-CG DNA duplex (Privalov and Crane-Robinson 2018b)

The situation with regard to unfolding the DNA duplex appears different from proteins as it is widely assumed that heat denaturation, i.e., strand separation, is not accompanied by any change in the heat capacity. This conclusion has been drawn from differential scanning calorimeter (DSC) studies of the heat denaturation process, as illustrated by the Cp/*T* function of a 12 bp all-CG duplex in Fig. 1. Linear extrapolation of the heat capacity function of the fully folded duplex (below 30 °C) coincides at high temperatures with the heat capacity of the fully unfolded state (above 100 °C). This appears to indicate that the heat capacities of the native duplex and the two separated strands are the same, a conclusion—if correct—of considerable convenience as it allows enthalpies determined for the melting of duplexes at different temperatures to be directly comparable. This simplification has resulted in tables of enthalpies/entropies of CG and AT pairs assumed valid for duplexes melting at different temperatures. Such tables are widely used, for example, in predicting the melting temperatures, *T*_m_, of the primers and probes used in PCR reactions.

**Fig. 2:**
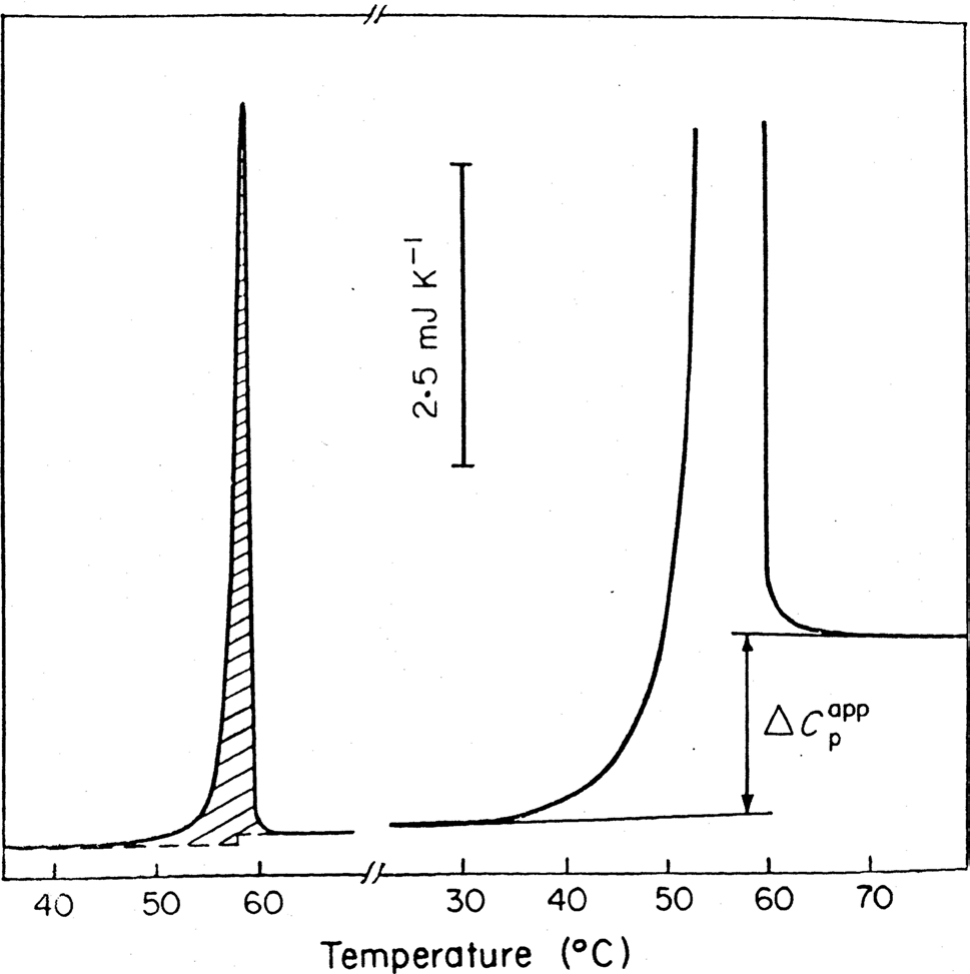
DSC recording of poly(A)∙poly(U) melting at 0.3 mM concentration (left) and a fragment of a recording at 5.0 mM concentration (right) The hatched area corresponds to the apparent melting enthalpy; arrows indicate the observed heat capacity change. The NaCl concentration is 0.1 M. (Filimonov and Privalov 1978). dΔ*H*^(A−U)^/d*T* = 134 ± 10 J K^−l^ mol^−l^

The continuing use of these data tables is somewhat surprising bearing in mind the evidence for a significant increase in Cp on duplex denaturation. Filimonov and Privalov 1978 demonstrated a significant increment for the melting of long poly(A)∙poly(U) molecules, and measured ΔCp as 134 ± 10 J K^−l^ mol-bp^−l^ (see Fig. 2). More recently, Chalikian et al. 1999, plotted the melting enthalpies of a broad range of double-stranded polynucleotides against their T_m_ values, to give a linear plot of slope *δ*(Δ*H*)/*δT* = 196 J K^−l^ mol-bp^−l^. Holbrook et al. 1999 derived values of ΔCp between 240 and 390 J K^−l^ mol-bp^−l^ for a 14 bp duplex of mixed composition, whilst Rouzina and Bloomfield 1999 indicated a range between 170 and 420 J K^−l^ mol-bp^−l^ for a broad set of genomic DNAs. In the same year, Jelesarov et al. 1999 used DSC measurements of residual structures in the separated strands to correct ITC determinations of the enthalpies of duplex formation and thereby derived *δ*(Δ*H*)/*δT* functions over a wide temperature interval to yield ΔCp values of about 200 J K^−l^ mol-bp^−l^. The question is then whether these earlier estimates are borne out by more recent measurements.

**Fig. 3:**
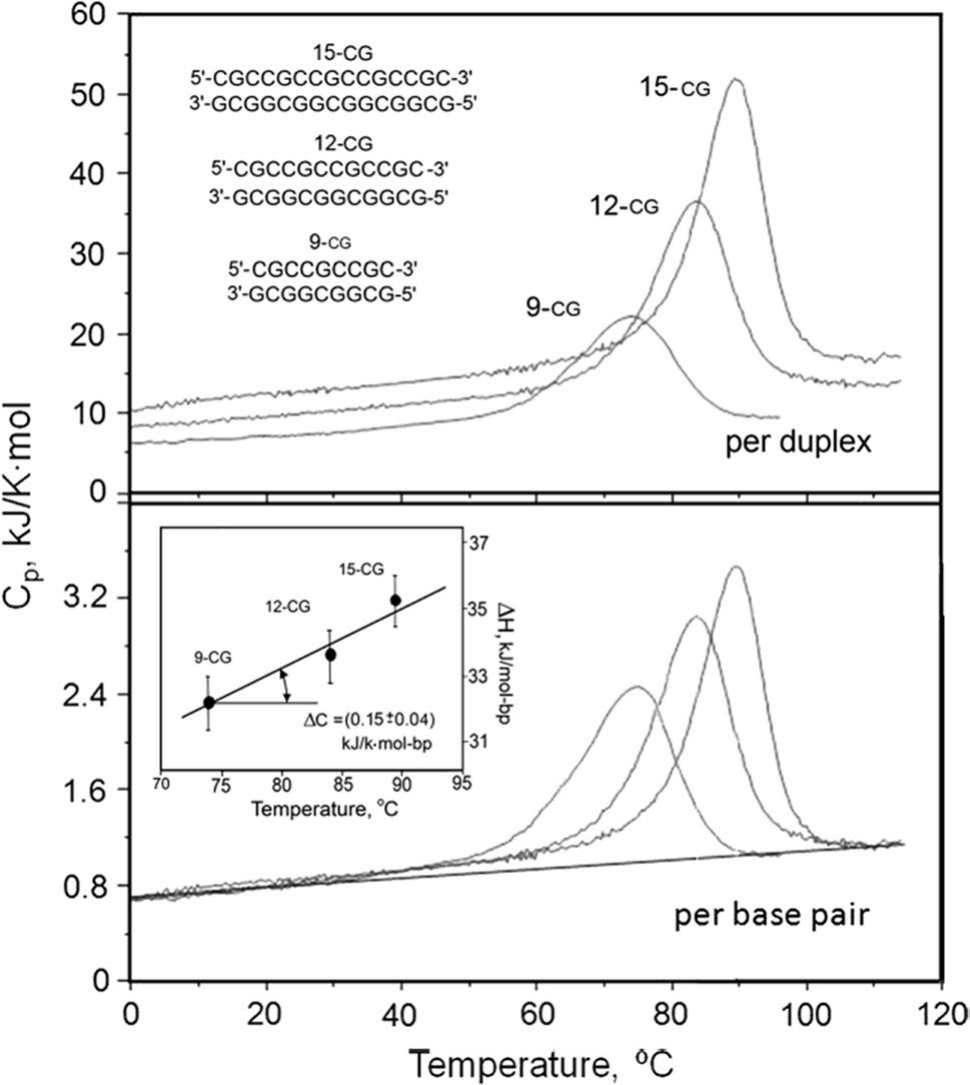
The partial heat capacity functions of three all-CG DNA duplexes calculated per mole of duplex (molar heat capacity, upper panel) and per mole of base pair (specific molar heat capacity, lower panel), all measured at the same molarity, 230 μM, of the duplexes in 150 mM NaCl, 5 mM Na-phosphate, pH 7.4. Inset: the dependence of the excess enthalpy on the transition temperature, the slope of which gives an estimate of ΔCp (Privalov and Crane-Robinson 2018b)

A closer look at the melting of all-CG duplexes of different lengths, and thus melting temperatures, indicates that the assumption of a zero ΔCp is indeed incorrect (Fig. 3). The upper panel shows the molar Cp/*T* functions for 9, 12 and 15-bp duplexes. As expected, the absolute heat capacities of the fully folded duplexes (at low temperatures) are in proportion to their lengths, as are their denatured states at high temperature—and the *T*_m_ values also increase with the length, as expected. If the Cp/*T* functions are plotted per base pair (i.e., the specific heat capacities) and a baseline is drawn on the assumption of a zero ΔCp value (as in Fig. 1), it turns out that the total enthalpy increases somewhat with temperature—as seen in the inset to the lower panel of Fig. 3. The slope of the Δ*H*/*T* plot, i.e., ΔCp, is about 0.15 kJ K^−1^ mol-bp^−1^.

**Fig. 4:**
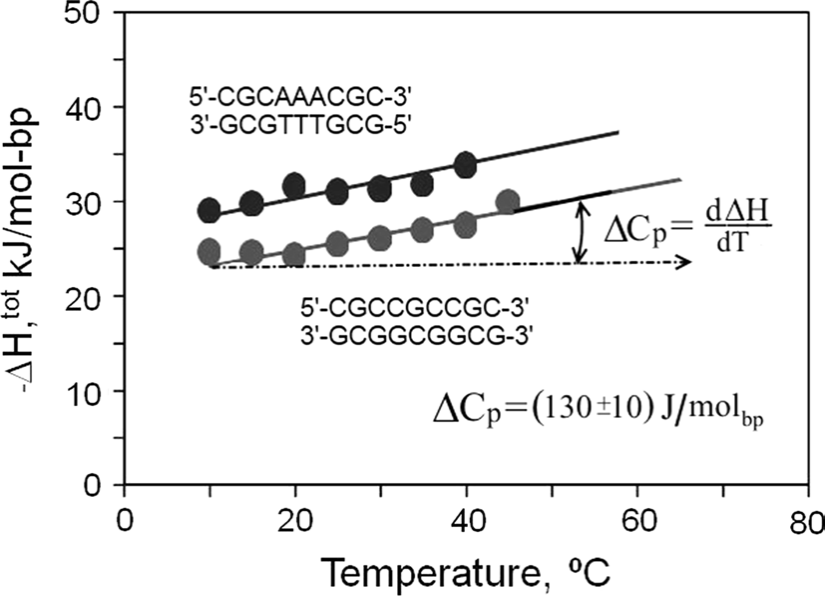
The ITC measured enthalpies of formation of two 9-bp DNA duplexes at various temperatures from 10 to 45 °C: one consisting only of CG base pairs, the other containing an A/T.A/T.A/T triplet (for more detail, see Vaitiekunas et al. 2015; Privalov and Crane-Robinson 2018b)

An alternative approach to verify the magnitude of ΔCp, without making any assumptions regarding the background appropriate for DSC scans, is to titrate one strand into its complement in the isothermal titration calorimeter (ITC). This has the advantage that experiments can be conducted over a wider temperature range, though the observed enthalpies require correction for residual structure in the individual stands at the temperature of each experiment (see Jelesarov et al. 1999; Vaitiekunas et al. 2015 for details). Figure 4 shows ITC-derived enthalpies for two 9-bp duplexes: one the 9-bp all CG duplex from Fig. 3 and the other of the same length but with the central 3 base pairs changed to AT. The enthalpies recorded for the AT-containing duplex are somewhat greater, because the heat of denaturing AT pairs is significantly greater than for CG pairs (Vaitiekunas et al. 2015) but the slope of the two functions is the same at 0.13 kJ K^−1^ mol-bp^−1^—a value which can be taken as the magnitude of ΔCp for both AT and CG pairs.

The heat capacity increase on DNA dissociation is thus positive but much lower in magnitude than for proteins. For comparison: ubiquitin (M.Wt. = 8.6 kDa), has ΔCp^25C^ ~ + 6 kJ K^−1^mol^−1^, whereas for the 12 bp DNA all-CG duplex (M.Wt. = 7.4 kDa), ΔCp^25C^ is measured as + (12 × 0.13) = + 1.56 kJ K^−1^ mol^−1^, i.e., the *specific* heat capacity change on unfolding is much less for the DNA duplex than for the protein.

**Fig. 5:**
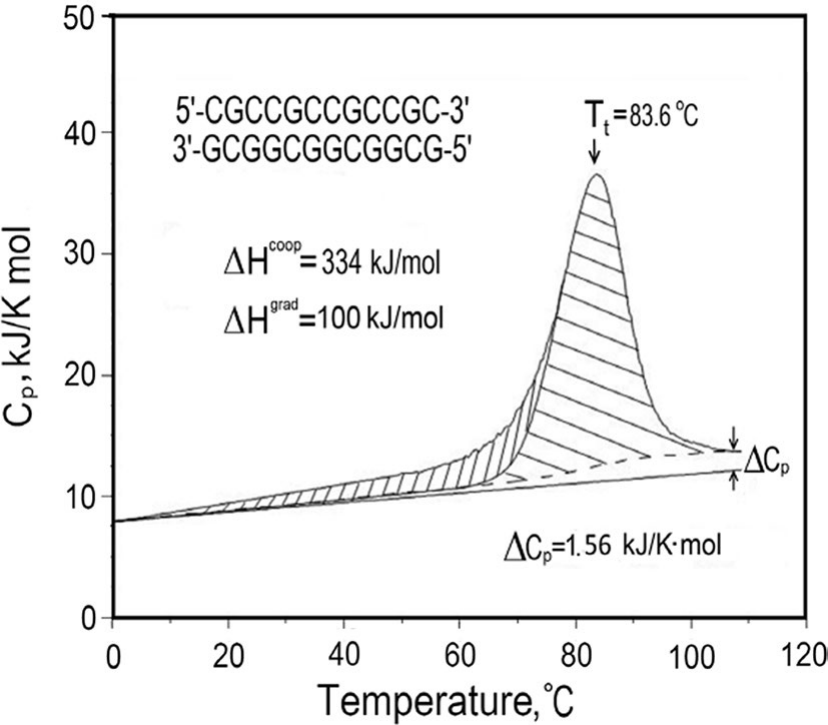
The observed heat capacity profile of a 12 bp all-CG duplex. The expected heat capacity of the fully folded DNA duplex is obtained by subtracting the heat capacity increment, ΔCp, (12 × 0.13 = 1.56 kJ K^−1^ mol^−1^), from the heat capacity of DNA at 110 °C and linearly extrapolating back to the start of melting at 0 °C. The experimental excess heat effect is then deconvoluted into non-cooperative (gradual, vertical hatching) and cooperative (horizontal hatching) phases (Vaitiekunas et al. 2015)

Determination of ΔCp for the base pairs of DNA has significant consequences for interpreting the Cp/*T* functions obtained in the scanning calorimeter. As explained in the caption to Fig. 5, it allows construction of a linear heat capacity function for the native, folded, state. This, in turn, allows the total excess heat to be deconvoluted into two components: the main peak that corresponds to a two-state cooperative dissociation process, preceded by a gradual accumulation of heat in the intact duplex structure. The enthalpy that characterizes the strand dissociation process, i.e., the melting, is that of the cooperative transition, not the total excess heat—as was previously assumed.

## Surfaces exposed upon DNA duplex dissociation

As the DNA duplex is a macromolecular complex with stacked aromatic bases located internally to the external polar phosphodiester chains, a situation not dissimilar to folded proteins, it is unsurprising that disruption of this structure is accompanied by an increase in the heat capacity—reflecting the exposure of apolar groupings to the solvent. Striking, however, is the fact that the magnitude of ΔCp is only about ¼ that of a protein of similar weight. Understanding this difference comes from measuring the magnitude of the two types of surface exposed on duplex denaturation, i.e., the increase in the polar and apolar accessible surface areas (ΔASAs in Eq. 1).

To quantify the contributions of polar and apolar contacts in the DNA duplex, the increases in accessible surface areas on strand separation were assessed using the Naccess program with two categories of surface atoms: polar (N, O and P) and apolar (C and H), see Table 1. For the folded forms, four B-form duplexes with high-resolution structures available and having mixed composition and variable length were selected. The question then arises as to the state of the single-stranded oligonucleotides that result from the heat dissociation process: can they be taken as totally randomised with full solvent access to the bases or does some secondary/tertiary structure remain? This is answered by DSC scans of individual single strands that demonstrate the presence of intrinsic structures at low temperatures that melt to yield linear Cp/*T* functions above 80/90 °C. Measurement shows that the slopes of these linear Cp/*T* functions—that represent the intrinsic heat capacity of the disordered chains—and also the absolute values of the heat capacities at 80/90 °C are in direct proportion to the length of the oligonucleotides (Jelesarov et al. 1999). This demonstrates that the heat denatured state is the same for all of such oligonucleotides—and is strongly suggestive that this is a fully disordered strand.

**Table 1:**
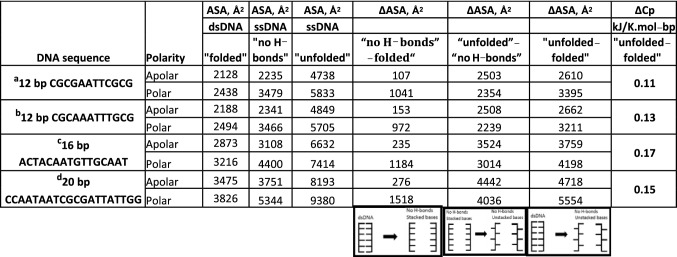
Water accessible surface areas (ASAs)—apolar and polar—and their changes for B-form DNA unfolding in two steps

*Step 1* native folded dsDNA to a hypothetical ssDNA state with broken H-bonds but the stacking within each strand preserved [LH cartoon]

*Step 2* the stacked ssDNA state to totally unfolded strands having complete solvent access to unstacked bases [central cartoon]. The RH cartoon depicts the aggregate of these two steps: the complete transition from folded dsDNA to totally unfolded strands. The total increases “unfolded–folded” (∆ASAs) are used to calculate the corresponding changes in the heat capacities, ∆Cp, using the equation of Makhatadze and Privalov (1990) and are given in the last column

*dsDNA* double-stranded DNA, *ssDNA* single-stranded DNA

^a^Drew et al. (1981), PDB:1BNA; ^b^Woods et al. (2004), PDB:1S2R; ^c^Narayana and Weiss (2009), PDB:3BSE. ^d^Garcia et al. (2016), PDB:5F9I

To model such randomly disordered and solvent-exposed strands, successive nucleotides in n-mer chains were spaced by (n − 1) abasic sites to ensure full access of the n bases to solvent. The Naccess programme does not recognize unnatural nucleotides, so the measured ASAs correspond to fully extended and exposed native oligodeoxynucleotide chains. Two checks of this assumption were made: (1) the abasic sites were removed manually—with no resulting change in ASA values—and (2) the chains were modelled by simple addition of the ASA values determined for the four individual mononucleotides: this resulted in an increase in the polar contribution by 1.4% and no change in the apolar contribution.

The duplex melting process was separated into two steps: Step 1 is separation of the two strands without altering their conformation, a process that represents breakage of the H-bonds (loss of pairing) without loss of base stacking. Table 1 shows that the increases in ASA in this step are largely polar (only ~ 14% apolar) as expected from exposure of the largely polar edges of the bases: this step represents loss of ~ 18% of the total contact area between the two strands. Step 2 is unstacking adjacent bases, i.e., randomising the separated polynucleotide chains, for which the total increase in ASA is much greater and is approximately equally divided between polar and apolar contributions: it represents the remaining 82% of the total contact area. The overall increase in accessible surface areas (“unfolded–folded”) averages to 45% apolar/55% polar. Previous measurements of changes in water accessible areas on DNA unfolding were reported by Holbrook et al. (1999) for a 14 bp duplex using the ANAREA programme. They noted that the base pairing interaction (‘helix to helix’) is almost completely polar—exactly as found here. However, the unstacking process (‘helix to disordered’) was found to be as much as 60% polar rather than the 47% polar measured here—although in both data sets, it is the unstacking process that makes the dominant contribution to the heat capacity change. When Holbrook et al.’s ΔASA values were substituted into the their own heat capacity function (Spolar et al. 1992), the increased negative contribution to ΔCp from hydration of polar surface was sufficient to fully negate the lower positive effect from the apolar term, i.e., a net zero ΔCp was predicted.

In contrast to DNA, the total increase in ASA for the unfolding of ubiquitin (5780 Å^2^) is 67% apolar/33% polar, figures typical for small globular proteins. This comparison demonstrates a key difference between the unfolding of DNA and proteins: dissociation of the DNA strands results in a much greater exposure of polar surface than is the case for proteins. For proteins, the dominant apolar component leads to the well-known positive values of ΔCp, but with DNA this is very much reduced in magnitude by the substantial negative contribution to ΔCp from the large polar ΔASA—despite its smaller ΔCp^*i*^ coefficient.

The last column of Table 1 gives the heat capacity changes calculated on the basis of the total apolar and polar ΔASAs using the above Eq. (2): the predicted values are close to the observed value of 0.13 kJ K^−1^ mol-bp^−1^ (Vaitiekunas et al. 2015). This correspondence demonstrates that the equation derived on the basis of unfolding the polypeptide chain (Eq. 2) applies effectively to polynucleotides and also supports the assumption that the fully denatured state used to model the ASA of the unfolded DNA strands effectively corresponds to the heat denatured state at high temperature. It is clear, therefore, that the heat capacity increase on melting the DNA duplex, although positive as for proteins, is much less in magnitude as a result of the large negative contribution from exposure of internal polar surface, rather than dominated by the apolar surface as for folded proteins.

## The significance of a heat capacity increase in DNA duplex dissociation

A heat capacity increase of 0.13 kJ K^−1^ mol-bp^−1^, i.e., 1.56 kJ K^−1^ for a 12-mer duplex, may seem of little consequence when compared to that of comparable globular proteins but when applied, for example, to calculation of the melting temperatures of PCR primers—a widespread use of DNA thermodynamic data—it is of important significance. For example, the denaturation enthalpy of a CG pair is about 19 kJ mol^−1^ at the standard temperature of 25 °C but extrapolation to, say, 75 °C (a typical primer *T*_m_) adds 6.5 kJ mol^−1^ to this, which is an increase of more than 30%!

Bearing in mind that the usual protocols for calculating the T_m_ of primers and probes for real-time PCR assume no variation of the enthalpy/entropy with temperature—and are thus fundamentally flawed—what, in practical terms, are the consequences of the observed ΔCp for *T*_m_ prediction? At present, ‘universal’ tables of characteristic enthalpies and entropies for the ten possible base pair adjacencies (the ‘nearest neighbour—NN—interactions’) and valid at all temperatures, are used to predict a wide range of *T*_m_ values. With a finite ΔCp, however, a completely different prediction protocol is required. The central issue is as follows: the enthalpy/entropy values assigned to CG and AT pairs for the standard temperature of 25 °C must be extrapolated to *T*_m_, the unknown we are attempting to calculate. This problem is best solved iteratively, rather than analytically: melting of short DNA duplexes takes place at temperatures between 50 and 95 °C, so to a first approximation a *T*_m_ of 75 °C can be used, i.e., 50° above the standard temperature, to calculate the enthalpies/entropies expected for that temperature—the ratio of which yields a postulated *T*_m_. This value of *T*_m_ can then be used for a second extrapolation. Usually the second iteration gives a predicted value of *T*_m_ that is not changed by further cycles. This protocol is given in detail in Privalov and Crane-Robinson (2018a).

A matter of interest is then to establish if the use of more accurate and temperature-dependent enthalpies/entropies leads to increased precision in predictions of the melting temperatures of short duplexes, for example the primers and probes of PCR reactions. Comparison was, therefore, made, for four duplexes, of their melting temperatures predicted using: (1) two of the well-known online calculators (OligoCalc, OC, from Northwestern University and OligoAnalyser, OA, from the company IDT) based on the NN tables of temperature-independent enthalpies/entropies published by Breslauer et al. (1986), Sugimoto et al. (1996) and SantaLucia (1998), and (2) the iterative protocol outlined above using temperature-dependent single-valued enthalpies/entropies for CG and AT pairs.

Table 2 shows substantial differences in the enthalpy/entropy values between those used in the PLP/CCR protocol and those from the ‘historical’ data sets: Δ*H*^PLP/CCR^ and Δ*S*^PLP/CCR^ values are significantly lower. This is largely a result of using just the cooperative component of the total enthalpy/entropy and also results from the fact that the enthalpy/entropy of AT pairs are in fact greater than those of CG pairs, as explained in Vaitiekunas et al. (2015). However, it is the ratio of the enthalpy to the entropy that determines *T*_m_, not their absolute values. Despite these large differences in the enthalpies and entropies, the predictive capacities of the two approaches do not differ greatly, with the notable exception of the 15-bp all-CG duplex, for which the PLP/CCR protocol is strikingly accurate: the difference of 0.4° in 362 K between the predicted and observed *T*_m_ represents an error of only 0.1%. The reason for such high precision is that the enthalpy/entropy for a CG pair is independent of its neighbour when that is also a CG pair, i.e., the predictive capability for all-CG duplexes is exceptionally high. This is not the case, however, for the enthalpy/entropy of an AT pair, which does depend on its neighbours, a variation that results from the presence of water tightly bound to AT pairs in the minor groove, but absent from all-CG duplexes. However, the current PLP/CCR protocol is based on single, unique values for the enthalpy/entropy of an AT pair, i.e., takes no account of NN interactions. The lack of correction for such interactions results in less accurate *T*_m_ predictions for the three AT-containing duplexes than for all-CG duplexes.

**Table 2:** Comparison of melting temperatures predicted by OligoCalc (OC) and OligoAnalyser (OA)—with *T*_m_ values obtained by the iterative methodology of PLP/CCR (Privalov and Crane-Robinson 2018a)

DNA duplexes	*T*_m_^obs^ (°C) in 0.15 M NaCl	∆*H*^OC^ (kJ mol^−1^)	∆*S*^OC^ (J K^−1^ mol^−1^)	*T*_m_^Predicted^ (°C) using	∆*H*^PLP/CCR^ (kJ mol^−1^)	∆*S*^PLP/CCR^ (J K^−1^ mol^−1^)	*T*_m_^Predicted^ (°C) using PLP/CCR
				OC OA			
5′-CGCCGCCGCCGCCGC-3′	89.5	648	1632	86.3	407	1124	88.9
3′-GCGGCGGCGGCGGCG-5′				91.2			
5′-CGCAAATTTAAACGC-3′	64.8	512	1347	64.9	416	1236	63.4
3′-GCGTTTAAATTTGCG-5′				63.7			
5′-CGCACACACACACGC-3′	75.9	554	1435	72.8	412	1189	73.3
3′-GCGTGTGTGTGTGCG-5′				75.1			
5′-GCGAACAATCGG-3′	64.8	426	1109	63.8	320	940	67.2
3′-CGCTTGTTAGCC-5′				64.7			

All duplex concentrations = 283 μM. Observed melting temperatures in column 2; Predictions in columns 5 and 8

The remaining point of interest is that despite the very substantial inaccuracies in the historical enthalpy/entropy data sets and the neglect of their temperature dependence, the OC and OA predictive capability is reasonably good: why is this? Firstly, it must be recalled that only the enthalpy is measured experimentally for the calibrating duplexes and the entropy is then derived by dividing this by the melting temperature, *T*_m_. As *T*_m_ prediction is the reverse process, errors in the predicted value are not great because inaccuracies in the calibrating enthalpies have been ‘compensated’ by corresponding errors in the entropies derived from them. Furthermore, since most of the predicted sequences melt at temperatures not so far from those of the calibrating duplexes, the importance of ΔCp for *T*_m_ prediction is reduced. It follows, therefore, that although the temperature-dependent enthalpies/entropies of the PLP/CCR protocol are much more appropriate than those in the historical data tables, further analysis of AT-containing duplexes is required to establish a precise NN interaction table and thereby bring the precision of all predictions up to the level already achieved for all-CG duplexes.

## Conclusions

Measurement of increases in solvent accessible surface areas (ASAs) as the B-form duplex dissociates into fully disordered single strands shows that these average to 45% apolar/55% polar surface. This distribution differs markedly from that of proteins for which the apolar surface exposed on denaturation amounts to about 2/3rd of the total. The core structure of the duplex is thus very much more dependent on polar interactions than is that of proteins. The immediate consequence of this is that the negative heat capacity effect of exposing the polar surface reduces the net increase in Cp for DNA to a low positive value. A finite ΔCp value for DNA implies that changes are required to the methods of calculating duplex melting temperatures.
